# Prevalence and management of antibiotic associated diarrhea in general hospitals

**DOI:** 10.1186/s12879-015-0869-0

**Published:** 2015-03-17

**Authors:** Monique M Elseviers, Yoleen Van Camp, Sander Nayaert, Khyra Duré, Lieven Annemans, Ann Tanghe, Sebastian Vermeersch

**Affiliations:** Centre for Research and Innovation in Care (CRIC), Faculty of Medicine and Health Sciences, University of Antwerp, CDE R3.29, Universiteitsplein 1, B-2610 Wilrijk, Antwerp Belgium; Department of Public Health, Interuniversity centre for health economics research (I-CHER), Ghent University, Ghent, Belgium; Hict, Health Economic Services, Brugge, Belgium

**Keywords:** Antibiotic use (AB), Antibiotic associated diarrhea (AAD), C*lostridium difficile* infection, AB use point prevalence, AAD prevalence, Contamination control, AAD related nursing care

## Abstract

**Background:**

Antibiotic-associated diarrhea (AAD) is a common adverse effect of antibiotic (AB) treatment. This study aimed to measure the overall prevalence of AAD (including mild to moderate diarrhea) in hospitalized AB treated patients, to investigate associated risk factors and to document AAD associated diagnostic investigations, contamination control and treatment.

**Methods:**

During 8 observation days (with time delay of 10–14 days between each observation day), all adult patients hospitalized at an internal medicine ward of 4 Belgian participating hospitals were screened for AB use. Patients receiving AB on the observation day were included in the study and screened for signs and symptoms of AAD using a period prevalence methodology. Clinical data were collected for all AB users and AAD related investigations and treatment were collected for the entire duration of AAD. Additionally, nurses noted daily the frequency of all extra care associated to the treatment of the diarrhea.

**Results:**

A total of 2543 hospitalized patients were screened of which 743 were treated with AB (29.2%). Included AB users had a mean age of 68 yr (range 16–99) and 52% were male. Penicillins were mostly used (63%) and 19% received more than one AB. AAD was observed in 9.6% of AB users including 4 with confirmed C*lostridium difficile* infection. AAD started between 1 and 16 days after AB start (median 5) and had a duration of 2 to 41 days (median 4). AAD was significantly associated with higher age and the use of double AB and proton pump inhibitors. AAD patients had extra laboratory investigations (79%), received extra pharmacological treatment (42%) and 10 of them were isolated (14%). AAD related extra nursing time amounted to 51 minutes per day for the treatment of diarrhea.

**Conclusions:**

In this observational study, with one third of hospitalized patients receiving AB, an AAD period prevalence of 9.6% in AB users was found. AAD caused extra investigations and treatment and an estimated extra nursing care of almost one hour per day. Preventive action are highly recommended to reduce the prevalence of AAD and associated health care costs.

## Background

In Europe, about one third of patients receives antibiotic (AB) therapy during hospitalization. Highest frequencies of AB treatment are observed in intensive care units and in surgical and internal medicine departments [1]. A common adverse effect of AB treatment is the development of antibiotic-associated diarrhea (AAD) with symptoms ranging from mild to severe attacks [2]. Most of the cases are benign and resolve under symptomatic treatment. Particularly if the diarrhea is associated with a *Clostridium difficile* infection, symptoms are more severe and can lead to a fulminant, relapsing and occasionally fatal colitis [3]. AAD, and particularly the more severe forms of *Clostridium difficile* infection, may result in increased diagnostic procedures, extended hospital stay and increased medical care costs [4,5].

The global prevalence of AAD, with inclusion of the mild to moderate attacks without further clinical diagnostic evaluation, is not well established. Attack rates vary depending on the antibiotic used, the epidemiological setting and the host [3]. Increased frequencies are found in children and advanced age. Additionally, underlying illness, recent surgery and drugs that alter bowel motility are factors that increase the risk of AAD development [2]. Reported prevalence ranges from 3.2 to 29.0%. Based on a recently published meta-analysis of RCTs investigating the value of probiotics for the prevention of AAD, we calculated a weighted prevalence of AAD of 14% in the control populations [6]. Among all AAD cases, 10 to 20% are associated with *Clostridium difficile* infection [7] resulting in a mean estimated incidence in Belgian hospitals of 0.91 per 1000 hospital admissions in 2011 [8].

Using the methodology of a point prevalence investigation to check for antibiotic use, this study aims to measure the period prevalence of AAD in hospitalized patients in the northern part of Belgium and to document the associated diagnostic investigations, contamination control and extra nursing care for the treatment of diarrhea.

## Methods

**Figure 1:**
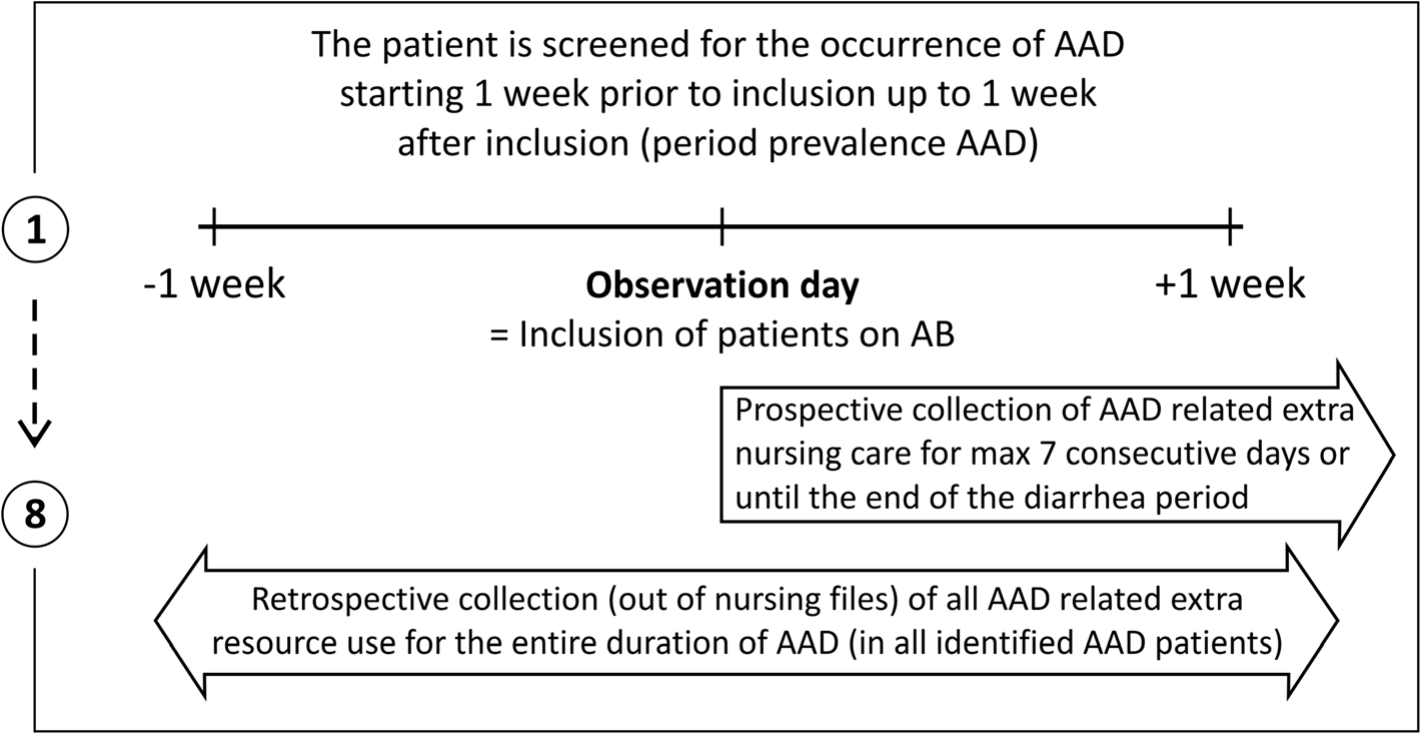
**Screening procedure for inclusion of antibiotic users (= point prevalence of AB use) and antibiotic associated diarrhea (= period prevalence of AAD).**

In all adult patients, hospitalized in one of the internal medicine wards of four participating hospitals, a point prevalence methodology was used to screen for AB use (Figure 1). Charts from all patients treated with AB on the observation day were investigated for signs and symptoms of AAD on that day as well as in the week before and the week after (period prevalence). In patients with AAD, related diagnostic procedures, contamination control, AAD treatment and extra nursing care were registered.

### Setting

One university hospital and three associated regional hospitals in the northern part of Belgium participated. Within these hospitals, all wards of the internal medicine department were included with exception of pediatric wards.

### Selection of patients

During the study period (January-April 2013), a research nurse visited all participating wards at time intervals of 10 to 14 day between observations. At each observation day, all hospitalized patients were screened for AB use. Patients were included in the study if over 16 years, hospitalized at the participating ward and treated with AB at the observation day (Figure 1).

### Sample size calculation

During the study period, an occupation rate of 20 patients per ward was expected. Given a total of 19 participating wards, about 380 patients could be screened for AB treatment at each observation day, resulting in a total of 2660 patients screened at the end of the study (i.e. after 8 observation days). With an expected frequency of 30% of patients treated with AB [1], 798 patients were expected to be included. Based on the estimation of 14% of patients that might develop AAD [6], the total number of AAD patients eligible for observations of AAD related diagnostic procedures and treatment would be 112. Using this sample size, the estimation of a 14% prevalence of AAD would have a 95% confidence interval of 11.6-16.4.

### Data collection

A patient checklist was used by the nurse researcher to collect data on personal characteristics (age, gender, ADL, dementia), hospitalization admission and discharge dates, AB treatment (start and stop date, type of AB prescribed) and clinical conditions known to increase the risk for the development of diarrhea (inflammatory bowel disease, diabetes, COPD, HIV, transplantation, chemotherapy, radiotherapy, endoscopy, abdominal surgery, use of proton pump inhibitors and nasogastric tubes) [2,3,9]. In case of diarrhea, start and stop dates were noted together with associated diagnostic procedures (laboratory tests, endoscopy, etc.), contamination control and diarrhea treatment (IV hydration, medication). AAD related investigations and treatments were collected for the entire duration of AAD.

Additionally, a checklist of nursing care for patients with diarrhea was completed by the treating nurses from the day of inclusion up to a maximum of seven consecutive days or until the end of the diarrhea period. Nurses noted per day of treatment the frequency of all extra care associated to diarrhea.

### Diagnosis of AAD

A chart review of the nursing files of all included AB patients, completed with information from the treating nurse, was performed aiming to identify signs and symptoms of diarrhea. In all included wards, it is part of the clinical practice to screen daily for diarrhea and to note the results of the screening in the patient’s nursing files. The period prevalence of AAD was estimated by screening the charts of patients for diarrhea at the day of inclusion (= the observation day) as well as in the week before the inclusion day and the week after the inclusion day (Figure 1). Date of start and discontinuation of AB treatment as well as date of first signs and end of diarrhea were carefully noted.

The diagnosis of AAD was based on the most commonly used definition found in literature [2,3,6,10]. Based on the collected data, patients were considered as having diarrhea if a change in normal stool frequency was observed with at least three loose or watery stools per day for at least two consecutive days. Additionally, start dates of AB use and the development of diarrhea were compared and the diagnosis of AAD was made when the first signs and symptoms of diarrhea occurred after the start of AB use.

### AAD related nursing care

We compiled a list of all possible extra nursing care actions related to diarrhea. The list was developed by the research nurses in collaboration with their colleagues, all working in the internal medicine department. Selected diarrhea related actions were: (1) assistance to go to the bathroom, (2) assistance for using a bedpan, (3) extra hygienic care, (4) replacement of bed linen, (5) replacement of incontinence material, (6) prevention of moisture injuries, (7) treatment of moisture injuries and (8) care for hydration.

Apart from the checklist used to register the frequency of extra nursing actions related to the care of diarrhea, a separate investigation was performed to estimate the working time needed to perform these extra actions. For this purpose 18 nurses working at internal medicine wards filled in a separate questionnaire with their estimation of the time needed to perform each of the registered actions. This data was analyzed by calculating the median, mean and trimmed mean time of care. Results were presented to an expert panel of 6 experienced nurses with a master degree and working in internal wards. Finally, standard time needed to perform each action was reached by consensus.

### Statistical analysis

Data analysis was performed using the statistical package IBM SPSS statistics, version 20.0 [11]. Mainly descriptive statistical methods were used to calculate the prevalence of AAD and to describe patient characteristics, associated risk factors, clinical course of diarrhea, diagnostic procedures and treatment of AAD patients. Differences in characteristics between AAD and non-AAD patients were analyzed using independent sample t-test (or Mann–Whitney-U test for skewed distributions) and chi-square test for means and proportions respectively. Logistic multiple regression analysis was performed to investigate risk factors associated with the development of AAD.

The time spent on diarrhea associated nursing care was calculated by multiplying the frequency registered for each action over the entire AAD observation period by the standard time for each action. The sum of total time spent to each action was divided by the number of observation days to obtain a daily mean time of AAD related nursing care. A level of significance of p < .05 and a confidence interval of 95% were used.

### Ethical considerations

Approval by the local ethical commission was given in January 2013 (EudractB009201216119/ECapproval4105s and B.U.N. 143201215730). Since inclusion of the total eligible population is a basic requirement for a trustful prevalence measurement, we had a long discussion with both ethical commissions to obtain a special permission to perform this study without informed consent. Permission was obtained under the condition that (1) the researchers had no any direct patient contact, (2) data were provided by the treating nurse based on the medical chart, and (3) data were completely anonymized before handed over to the researcher.

## Results

### Description of the study population

In the 19 participating wards of internal medicine, a total of 2543 hospitalized patients were screened and in 743 AB use was registered at the observation days, revealing a point prevalence of 29.2%. Included AB users had a mean age of 67.7 years (range 16–99), 51.5% were male and patients were hospitalized for a median of 15 days (range 1–238). Penicillins were mostly prescribed (see Table 1). In about half of the patients, antibiotic treatment was initiated for a respiratory tract infection. Only 4 out of 743 AB users (0.5%) received an additional prescription of probiotics as prevention. AB treatment had a median duration of 6 days (mean 7.3; range 2–41).

**Table 1:** **Description of the population**

	**Total sample of AB users n = 743**	**Comparison between nonAAD and AAD patients**		
		**nonAAD n = 672**	**AAD n = 71**	***p*** **value of difference**
**Demographics characteristics**				
Age (yrs) mean (range)	67.7 (16-99)	67.3 (18.1)	71.9 (16.8)	0.040
Gender% male	51.5%	47.9%	53.5%	0.369
**Clinical characteristics**				
Transfer from other ward	16.5%	16.4%	16.9%	0.917
ADL score (6-24) mean (SD)	12.3 (5.7)	12.0 (5.6)	14.2 (5.8)	0.002
Disorientation score (2-8) mean (SD)	2.9 (1.7)	2.9 (1.6)	3.5 (1.9)	0.001
**Risk factors for diarrhea**				
Inflammatory bowel disease	7.3%	7.4%	5.6%	0.577
Proton pump inhibitors	54.6%	53.1%	69.0%	0.011
Chemotherapy	6.2%	6.0%	8.5%	0.406
Radiotherapy	2.0%	1.8%	4.2%	0.165
Tube feeding	3.9%	3.6%	7.0%	0.151
Endoscopic procedures	15.9%	14.9%	25.4%	0.022
Abdominal surgery	3.1%	2.8%	5.6%	0.194
Diabetes	26.6%	25.7%	35.2%	0.086
COPD	25.4%	25.7%	22.5%	0.555
HIV	1.2%	1.2%	1.4%	0.873
Transplantation	3.0%	3.0%	2.8%	0.940
Decubitus	0.4%	0.1%	2.8%	0.001
Laxatives	15.8%	16.4%	10.9%	0.260
Risk score (sum of factors) mean (SD)	1.5 (1.1)	1.5 (1.1)	1.9 (1.1)	0.001
**Antibiotic use before diarrhea**				
More than one antibiotic prescribed	19.2%	18.0%	31.0%	0.008
Type of antibiotics*				
Penicillins	63.1%	62.9%	64.8%	0.760
Quinolones	22.2%	21.6%	28.2%	0.204
Cephalosporins	11.8%	12.1%	9.9%	0.586
Macrolides	8.1%	8.2%	7.0%	0.737
Aminoglycosides	3.4%	3.6%	1.4%	0.336
Sulfonamides	1.7%	1.8%	1.4%	0.818
Other ab	7.3%	7.0%	9.9%	0.376

*Type of antibiotics > 100% due to double and triple use.

### Prevalence of antibiotic associated diarrhea (AAD)

**Figure 2:**
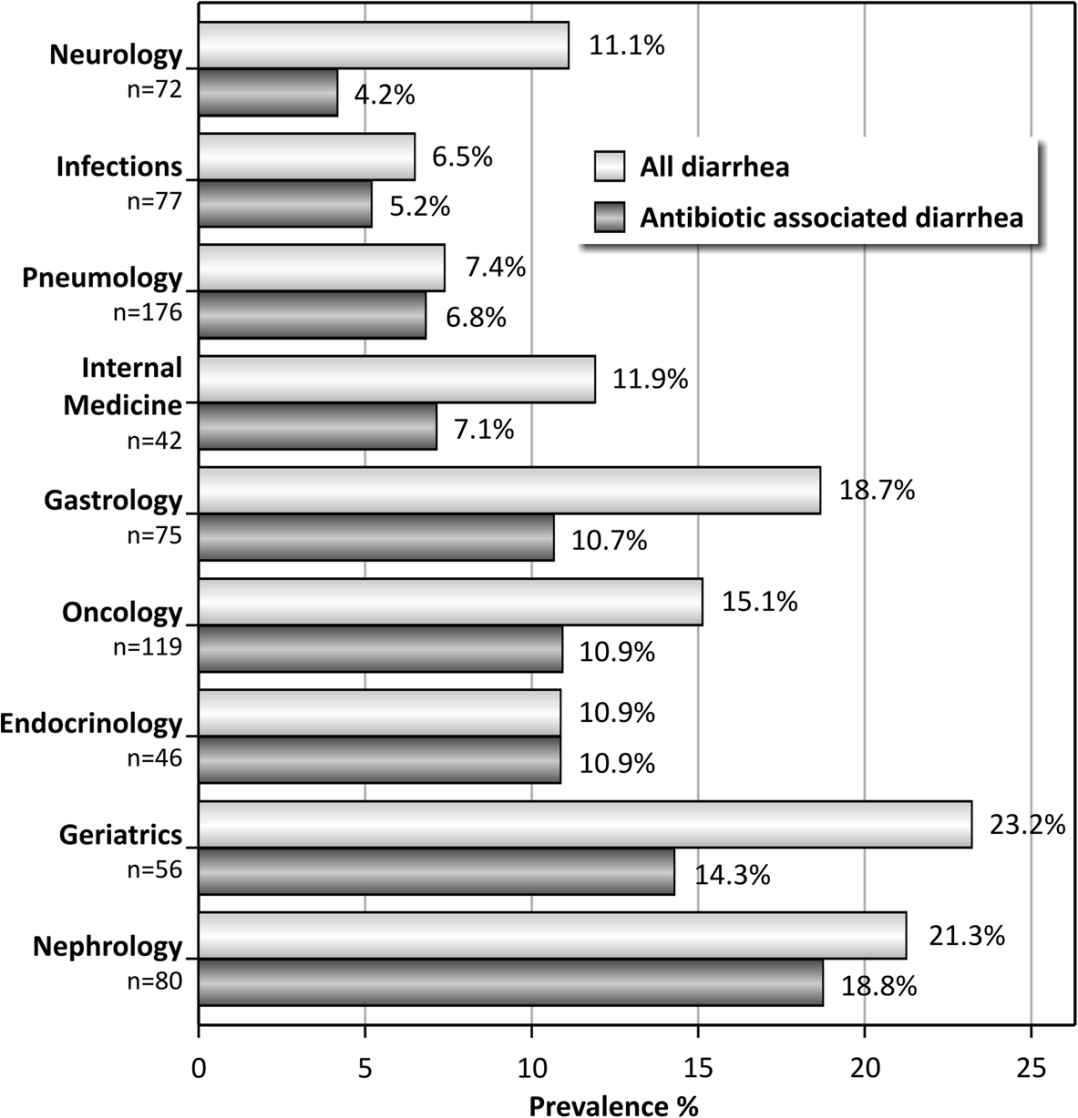
**Period prevalence of diarrhea and antibiotic-associated diarrhea (AAD) in hospitalized patients with antibiotic treatment (n = 743) according to the ward of admission.**

**Figure 3:**
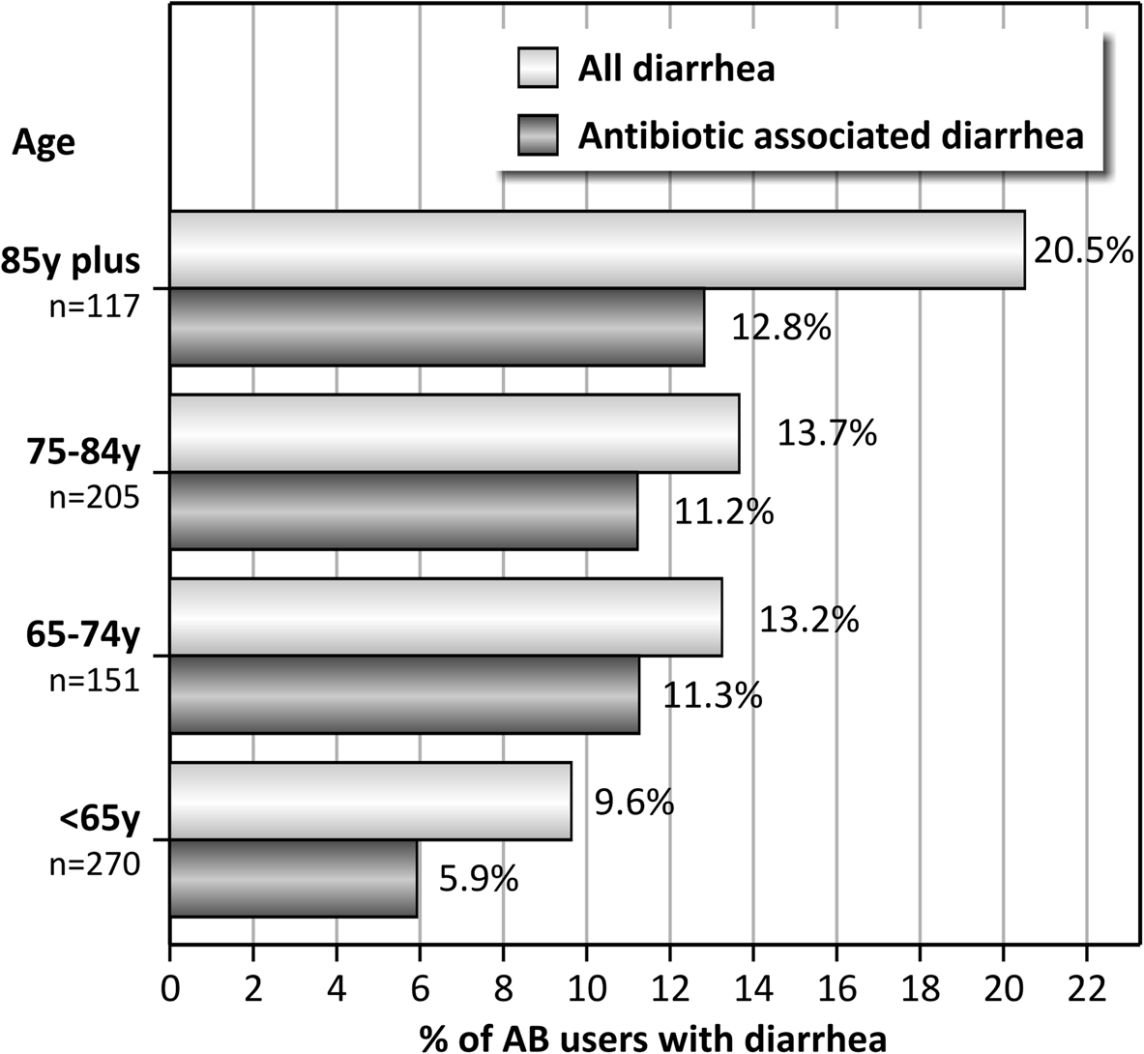
**Period prevalence of diarrhea and antibiotic-associated diarrhea (AAD) in hospitalized patients with antibiotic treatment (n = 743) according to age categories.**

In 98 of the 743 included AB users, signs and symptoms of diarrhea were noted (13.2%). Diarrhea developed after the start of AB treatment in 71 of them, giving a period prevalence of AAD of 9.6% (95% CI = 7.5-11.9%). The observed AAD prevalence varied between 4.2% in a ward of neurology to 18.8% in a ward of nephrology. Particularly in the wards of neurology, gastroenterology and geriatrics, large differences were observed between the prevalence of diarrhea from all causes and AAD (Figure 2). AAD prevalence varied also considerably between different age categories ranging from 5.9% in patients younger than 65 to 12.8% in patients over 85 (Figure 3). First signs and symptoms of AAD were observed between 1 and 16 days after the start of AB treatment (median 5). A large variation of antibiotic agents was involved in the development of AAD. The duration of AAD varied between 2 and 41 days (median 4; mean 4.9). Four patients were confirmed to have a *Clostridium difficile* infection with a median duration of diarrhea of 10 days (mean 11,0; range 10–13).

### Comparison between non-AAD and AAD patients

Patients with AAD were older, used more different antibiotics, had more problems with activities of daily living (higher ADL score), showed more disorientation in time and place (higher disorientation score) and had more risk factors associated with the development of AAD compared to non-AAD patients (see Table 1, comparison between AAD and non-AAD patients). Particularly a significant higher use of proton pump inhibitors, endoscopic procedures and decubitus was found in AAD patients. The type of antibiotics used by AAD and non-AAD patients did not differ.

Patients with AAD had a significant (p = 0.008) longer hospital stay compared to non-AAD patients with a median (range) of 21(5–122) and 14(1–238) days, respectively.

### Characteristics associated with the development of antibiotic associated diarrhea

In the univariate analysis, several demographic and clinical patients’ characteristics as well as previously reported risk factors and a particular type of ward were associated with the development of AAD (see Table 2). In the multivariate analysis the following characteristics were identified as independent risk factors for AAD: increased age, using more than one AB, increased ADL and disorientation scores, use of proton pump inhibitors, presence of decubitus and being hospitalized at a ward of nephrology. These factors had a limited Nagelkerke r square of 0.103 in the explanation of the variation in AAD development.

**Table 2:** **Risk factors associated with the development of AAD**

	**Univariate**	Multivariate*
**Associated factors**	**OR (95% CI)**	OR (95% CI)*
Age > 70y	2.22 (1.30-3.77)	2.41 (1.39-4.18)
More than one AB	2.05 (1.19-3.51)	2.27 (1.30-3.98)
ADL score (6-24)	1.07 (1.02-1.11)	
Disorientation score (2-8)	1.23 (1.09-1.39)	
Risk score (sum of risk factors)	1.42 (1.14-1.75)	
Proton pump inhibitors	1.97 (1.16-3.32)	1.98 (1.15-3.43)
Endoscropic procedures	1.94 (1.09-3.45)	
Diabetes	1.57 (0.94-2.63)	
Decubitus	19.45 (1.74-217.24)	32.11 (2.82-366.13)
University Hospital	1.70 (1.03-2.80)	
Nephrology	2.50 (1.34-4.67)	2.34 (1.23-4.47)

*Nagelkerke Rsquare = 0.103.

### Investigation of antibiotic associated diarrhea

In 79% of AAD positive patients, a baseline bacteriological investigation was performed with 23 patients receiving a standard culture (2 positive results) and 48 patients receiving a first specific investigation for *Clostridium difficile* (4 positive results). Additional standard tests were performed in 4 patients. Additional tests *for Clostridium difficile* were performed in 13 patients (all with negative test results during the baseline investigation) with 4 even having a third and 1 with a fourth test. All these additional investigations tested negative for *Clostridium difficile* (Table 3).

**Table 3:** **AAD related outcome and actions**

**AAD related investigations**	**n = 71**
Standard bacteriological investigations (n)	27
Specific for clostridium (n)	66
Other bacteriological investigations (n)	6
Additional investigations (endoscopy) (n)	1
**AAD related treatment**	**n = 71**
Patient isolation in single room (n)	10
Pharmacological treatment	
-Probiotics (enterol) (n)	9
-Antidiarrheal (loperamide) (n)	9
-Antibiotics (n)	1
-Antiparasitics (flagyl) (n)	3
IV hydration (n)	12
**AAD related nursing care**	
Extra daily nursing care time median (range)	51.3 (5-154)

### Treatment of antibiotic associated diarrhea

Only one of the AAD positive patients received an extra diagnostic investigation (endoscopy). Patient isolation (all with transfer to a single room) was applied in 10 patients (14% of AAD patients), including those four patients tested positive for *Clostridium difficile*. The median duration of isolation was 10 days. Pharmacological treatment of AAD was applied in 19 patients (27% of AAD patients) and consisted in the discontinuation of the original AB treatment (3 patients) and the prescription of probiotics (9 patients), antidiarrheals (9 patients), antibiotics (1 patients) and antiparasitic products (3 patients). Additionally, 11 patients received IV hydration (Table 3). Among the four patients with *Clostridium difficile* infection, pharmacological treatment was limited to one patient receiving an antiparasitic product, one patient receiving one antiparasitic and one antibiotic product and one patient receiving IV hydration.

### Extra nursing care related to antibiotic associated diarrhea

**Figure 4:**
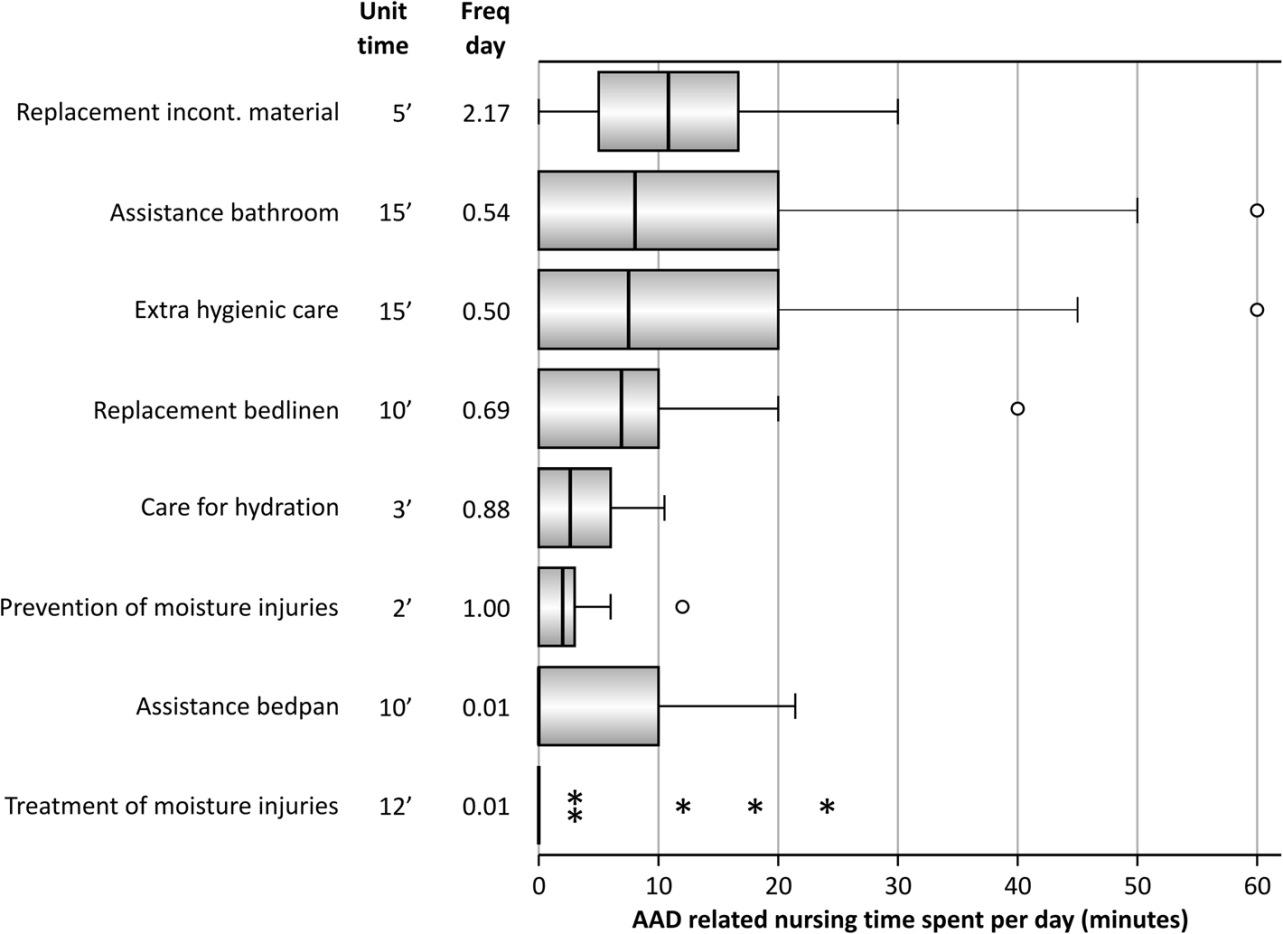
**Extra nursing care related to the treatment of patients with antibiotic-associated diarrhea (AAD) expressed as total time spent per action per day.** Frequency and estimation of nursing time spent to each action was based on observations in 26 AAD positive patients spread over 94 AAD days. Unit time = estimated time (in minutes) needed to perform this action. Freq day = median frequency of the action performed per patient and per day.

Observations of diarrhea related care were performed in 26 patients spread over 94 days with diarrhea. Patients included in this observation were somewhat older with a mean age of 75 (range 49–93), had a longer length of stay with a mean of 20 days (range 4–49) and had a longer period of diarrhea with a mean of 10 days (range 2–41). The standard time needed to perform each of the extra diarrhea related actions ranged from 3 minutes for the care of hydration to 15 minutes for assistance to go to the bathroom and for extra hygienic care (Figure 4). The total time spent to deliver the extra nursing care related to diarrhea treatment amounted to a median of 51 minutes per day (range 5–154). As shown in Figure 4, the most frequent extra action performed was the replacement of incontinence material with a median of 2.2 replacements per day (range 0–6). The replacement of incontinence material was also the most labor intensive with a median time spent of 10.8 minutes per day (range 0–30).

## Discussion

Our study revealed that one out of three patients hospitalized in internal medicine departments received AB treatment. Of these 743 AB users, 9.6% developed diarrhea that could be associated to AB use. Half of the patients with diarrhea underwent biological testing for *Clostridium difficile* (4 positive) and 10 patients were isolated.

### Strengths and limitations

A literature research revealed that reported incidences of AAD were undetermined showing a wide variation between population studies and clinical trials (reviewed by McFarland, 2008) [2]. The main contribution of this study is that our primary objective was to determine the overall prevalence of AAD (including uncomplicated cases) in all patients hospitalized in internal medicine departments and treated with AB. Moreover, we used a methodology enabling to distinguish patients being treated with antibiotics for diarrhea and patients developing diarrhea after the start of antibiotic treatment. By carefully documenting the start time of AB treatment and first symptoms of diarrhea, we had to exclude 27 patients (out of 98) with diarrhea from the AAD group since first signs of their diarrhea were observed before the start of AB treatment.

Based on control groups of clinical trials included in a recent review by Hempel and colleagues [6], we calculated a weighted prevalence rate of 14%. In our AAD study we found a lower prevalence of 9.6%. There are several reasons to think that our study slightly underestimated the real prevalence of AAD. First, our observations for the detection of diarrhea were limited to seven days post inclusion, while first signs and symptoms might have occurred later on. Second, we found a slightly higher prevalence in the university hospital where an electronic patient chart was used with the requirement to register nursing related problems per shift. It might be that this rigorous electronic registration system enabled faster and more complete registration of diarrhea (compared to the handwritten chart in the other hospitals), particularly in case of mild symptoms over a limited time period.

Most health economic studies investigating the health care costs associated with AAD, concentrated on *Clostridium difficile* associated cases [4,5]. Positive in our study was to focus on all AAD patients and to study extra nursing care for the treatment of diarrhea. Our observation that caring for a patient with diarrhea is rather labor intensive (amounting to 51 extra minutes of care per day) seems worthwhile to take into account in future health economic evaluations of AAD. Limitations were that (1) we only estimated the time spent per day for this extra treatment (not including cost calculations or extra requirements for patient isolation), and that (2) observations were limited to patients showing clear signs and symptoms of diarrhea at the observation day. As a result, patients included in this subsample had a longer median duration of diarrhea compared to patients in the complete sample.

### General discussion

Our point prevalence measurement of 29.7% of AB users is comparable with the results of the ESAC survey in European hospitals using the same methodology [1]. Based on a sample of 5 hospitals per country and including 37352 admitted patients in the ESAC project, a global point prevalence of AB treatment of 28.6% was registered, increasing to 29.8% in internal medicine and to 58,3% in intensive care units. For Belgium, ESAC reported an overall point prevalence of 27.7% in 2009.

In our sample, 4 patients screened positive for *Clostridium difficile*. Comparing this figure proved difficult as published rates are calculated in a variety of ways, using different denominators. In the review of McFarland [2], incidence rates for *Clostridium difficile* associated diarrhea ranged from 3.5/10000 to 18.5%. Asha and colleagues started from a sample of fecal specimens systematically tested for *Clostridium difficile* cytotoxins and reported a prevalence of 12.7% [9]. On the other hand, the Belgian Scientific Institute for Public Health found an incidence of 0.91 per 1000 hospital admissions based on collected reports of collaborating Belgian hospitals [12]. Depending on the denominator, we can present the incidence of the 4 positive patients of our study as 0.16% (4/2,543 patients screened during the observation days), 0.54% (4/743 patients with AB at the observation days) or 5.63% (4/71 patients with AAD). A more uniform system to report incidences with clear information about the denominator used is highly recommended in this domain.

Several health economic studies focused on the health care costs related to *Clostridium difficile* associated diarrhea showing that these patients had a significant increase in hospital costs mainly associated to a longer length of stay [4,13,14]. Dubberke and colleagues pointed to the additional increased health care costs observed 6 months after the initial hospitalization period [5]. In health economic studies, the costs associated to the much more prevalent but less severe AAD cases were not included. In our study, we took a first step to this calculation with the estimation of the extra nursing time spent for the treatment of AAD patients enabling to calculate associated extra costs for nursing care and material in a later phase of our study. In the study we also observed differences in the total hospital length of stay in AAD and non-AAD patients. Among AAD patients, those with a *Clostridium difficile* infection had a median hospitalization of 43.5 days (range 24–51). These differences in length of stay are not corrected for any confounding factors and needs further analysis.

Our study showed that probiotics were very rarely used for the prevention of AAD with only 4 out of 743 AB users (0.5%) receiving a probiotic treatment before the occurrence of diarrhea. The use of probiotics (particularly the non-pathogenic yeast *Saccharomyces boulardii*) for the prevention of AAD gained increasing attention in recent years [10,15]. Meta-analyses of studies focusing on the prevention of AAD in general revealed that preventive treatment with *Saccharomyces boulardii* halved the risk of AAD development [6,16,17]. In the light of these promising results, a cost-effectiveness analysis of preventive probiotic use in AB users is highly recommended to support a more generalized use of probiotics in clinical practice.

## Conclusions

In this study, with one third of hospitalized patients receiving AB treatment, an AAD period prevalence of 9.6% in AB users was found. AAD was associated with extra investigations, extra treatment and extra time of nursing care of almost one hour per day. Preventive actions are highly recommended to reduce the prevalence of AAD and associated health care costs.
